# A *S*-adenosylmethionine methyltransferase-like domain within the essential, Fe-S-containing yeast protein Dre2

**DOI:** 10.1111/j.1742-4658.2012.08597.x

**Published:** 2012-06

**Authors:** Nicolas Soler, Constantin T Craescu, Jacques Gallay, Yves-Michel Frapart, Daniel Mansuy, Bertrand Raynal, Giuseppe Baldacci, Annalisa Pastore, Meng-Er Huang, Laurence Vernis

**Affiliations:** 1 CNRS UMR2027, Centre UniversitaireOrsay, France; 2 Institut Curie, Centre de Recherche, Centre UniversitaireOrsay, France; 3 INSERM U759, Centre UniversitaireOrsay, France; 4 Institut de Biochimie et de Biophysique Moléculaire et Cellulaire, Université Paris-SudOrsay, France; 5 CNRS UMR8601, Université Paris DescartesSorbonne Paris Cité, France; 6 Institut Pasteur, Plateforme de Biophysique des Macromolécules et de leurs InteractionsParis, France; 7 Division of Molecular Structure, National Institute for Medical ResearchLondon, UK

**Keywords:** Dre2, iron-sulfur cluster, NMR, SAM methyltransferase fold, yeast

## Abstract

Yeast Dre2 is an essential Fe-S cluster-containing protein that has been implicated in cytosolic Fe-S protein biogenesis and in cell death regulation in response to oxidative stress. Its absence in yeast can be complemented by the human homologous antiapoptotic protein cytokine-induced apoptosis inhibitor 1 (also known as anamorsin), suggesting at least one common function. Using complementary techniques, we have investigated the biochemical and biophysical properties of Dre2. We show that it contains an N-terminal domain whose structure in solution consists of a stable well-structured monomer with an overall typical *S*-adenosylmethionine methyltransferase fold lacking two α-helices and a β-strand. The highly conserved C-terminus of Dre2, containing two Fe-S clusters, influences the flexibility of the N-terminal domain. We discuss the hypotheses that the activity of the N-terminal domain could be modulated by the redox activity of Fe-S clusters containing the C-terminus domain *in vivo*.

## Introduction

Dre2 is an essential yeast protein whose biological function is not yet fully understood. Preliminary analyses have indicated that Dre2 might be required in cases of defective DNA replication because a *dre2* mutated allele displays synthetic lethality with a mutated allele of *pol3* that encodes the catalytic subunit of DNA polymerase delta [1], and in chromosome segregation [2]. Further work identified that Dre2 is a Fe-S-containing protein with a role in cytosolic Fe-S protein biogenesis [3]. Dre2 forms a stable cytosolic complex with Tah18, which is an essential reductase exhibiting binding motifs for FMN, FAD and NAD cofactors. The Dre2-Tah18 complex has been implicated in the response to high levels of oxidative stress and the control of hydrogen peroxide-induced cell death [4]. According to this model, the cytoplasmic Dre2-Tah18 complex might act as a biosensor of high oxidative stress levels and control cell death in a mitochondria-dependent fashion. The Fe-S-containing C-terminus of Dre2 was demonstrated to receive electrons from the reductase Tah18, and the Dre2-Tah18 complex was positioned early in the process responsible for inserting Fe-S in target proteins [5]. Independently, genetic studies have further implicated Tah18 in DNA repair or/and replication [1,6,7].

Dre2 contains a highly-conserved C-terminus carrying the essential functions of Dre2 and containing two Fe-S clusters [3,5,8], and a less conserved N-terminus (1–172), which is not essential for yeast viability [8]. Sequence similarities were identified between the Dre2 C-terminal domain and the human protein Ciapin1 (cytokine-induced apoptosis inhibitor 1) (also known as anamorsin), indicating that these proteins are homologues. This last protein, which exhibits antiapoptotic activity both in human and murine cell lines [9], can efficiently replace Dre2 in yeast, indicating that the two proteins share at least one common function [3,4]. The human Tah18 orthologue Ndor1 associates with Ciapin1 and forms the yeast Dre2-Tah18 counterpart in human cells [5].

We performed a detailed structural study of the yeast Dre2 protein aiming to obtain further insights into its function. Using *in silico* and *in vitro* biophysical and biochemical analyses, we show that the Dre2 N-terminus [Dre2 (1–133)] is a stable, well-structured monomer. The 3D structure of Dre2 (1–133) in solution reveals close similarities to *S*-adenosylmethionine (SAM) methyltransferases, even though it lacks two α-helices and one β-sheet. Our data suggest that the presence of the Fe-S clusters influences the flexibility and the overall fold of the protein, particularly of the N-terminal SAM-methyltransferase related domain, possibly influencing its activity. We discuss the possibility that Dre2 might belong to the SAM radical superfamily of proteins, comprising enzymes that utilize Fe-S clusters and S-adenosylmethionine to initiate radical reactions.

## Results

### Sequence analysis of Dre2 predicts a stable, well-structured N-terminal domain

**Fig. 1:**
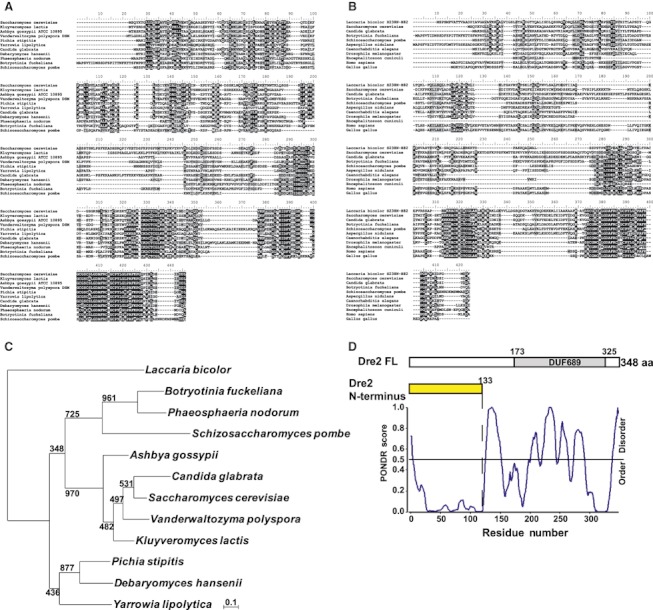
Dre2 N-terminus is conserved among Ascomycetes but not among Eucaryotes and is predicted to be a stable and structured domain. Multiple sequence alignment of Dre2 with homologues among Ascomycetes (A) and among Eucaryotes (B) using muscle software [25]. (C) Maximum likelihood tree of Dre2 homologues among Ascomycetes. Eleven Ascomycetes sequences and one Basidiomycetes sequence (Laccaria, as an external group) were aligned by muscle and 138 homologous positions were selected for tree calculation by phyml [28]. The robustness of the tree was estimated by nonparametric bootstrap analysis (1000 replicates). The scale bar represents the number of substitutions per site. (D) Predictions of intrinsically unstructured regions using pondr software. A block diagram of the architecture of Dre2 is shown at the top with the identification of the DUF689 motif, which includes two Fe-S clusters that receive electrons from the reductase Tah18. For Dre2 (1–133) purification, see Fig. S1.

To characterize full-length Dre2 *in silico*, we performed primary sequence alignments and secondary structure predictions. Database searches revealed that Dre2 has no paralogue in *Saccharomyces* *cerevisiae,* in agreement with its essential role. The Dre2 N-terminus is not highly conserved except in Ascomycetes (Fig. 1A,B). A phylogenetic analysis of Dre2 homologues among Ascomycetes showed that the evolution of the protein follows the evolution of organisms, in accordance with the fact that the protein is essential and that no lateral transfer occurred recently (Fig. 1C). By contrast, the C-terminus exhibits a high level of conservation and is widely spread within eukaryotic genomes. Searches for specific motifs gave no significant hits, except for a DUF689 motif in the C-terminus (DUF: domain of unknown function according to the PFAM database; recently renamed as IPR007785 Anamorsin). Eight of the nine cysteines in Dre2 protein are located in the DUF689, and they are likely to be involved in the coordination of previously described iron-sulfur clusters [3]. Fold analysis software (pondr, http://www.pondr.com; foldindex, http://bip.weizmann.ac.il/fldbin/findex) suggests the presence of intrinsically unfolded regions in the C-terminus of Dre2 and of a well-structured N-terminal domain (Fig. 1D).

### Experimental characterization of Dre2 N-terminus fold and stability

**Fig. 2:**
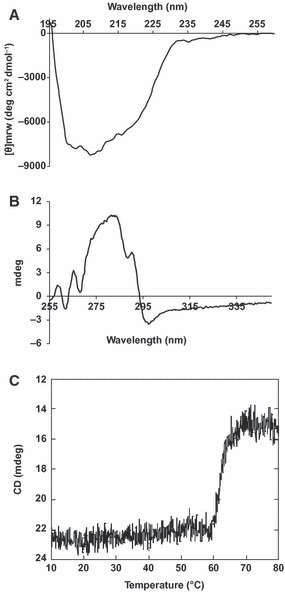
Experimental characterization of Dre2 N-terminus folding. (A) Far-UV CD spectrum of 10 μm Dre2 (1–133) at 10 °C. (B) Near-UV CD spectrum (250–320 nm) of 45 μm Dre2 (1–133) protein at 10 °C. (C) Thermal denaturation curve of Dre2 (1–133) at 291.8 nm from 10 to 80 °C (1 °C·min^−1^).

In agreement with the bioinformatics analysis, we expressed a construct spanning the sequence of the Dre2 N-terminus [Dre2 (1–133)] in *Escherichia coli* and purified this protein fragment for further structural characterization. The 15-kDa recombinant domain efficiently over-expresses as a soluble protein, which could be purified to homogeneity, as judged by Coomassie blue staining (Fig. S1). We first characterized Dre2 (1–133) by circular dichroism (CD), a method that allows experimental evaluation of the secondary and tertiary structure content [10]. The far-UV CD spectrum of Dre2 (1–133) is typical of an α/β content [10] (Fig. 2A). The spectrum shows an unusual but highly reproducible behaviour that is concentration-independent, with an unusual band around 202 nm that was attributed to an aromatic contribution. Dre2 (1–133) contains several aromatic residues (F36, 40, 73, 107; Y4, 57, 62, 98, 115; and W117). This hypothesis was supported by the near-UV CD spectrum (Fig. 2B), which also indicates a stable 3D structure.

Thermal denaturation followed at 222 nm (a wavelength with minimum ellipticity typical of α-helical structures) showed a highly cooperative transition with a midpoint at 62 °C (Fig. 2C) that is indicative of a stably-folded protein. Approximately 30% of the ellipticity was not recovered after cooling back to 10 °C (data not shown), indicating that the thermal denaturation is only partially reversible.

Taken together, these data indicate that Dre2 (1–133) is a well-structured domain and thus the determination of the three-dimensional structure by NMR is in principle feasible.

### Dre2 (1–133) is monomeric

**Fig. 3:**
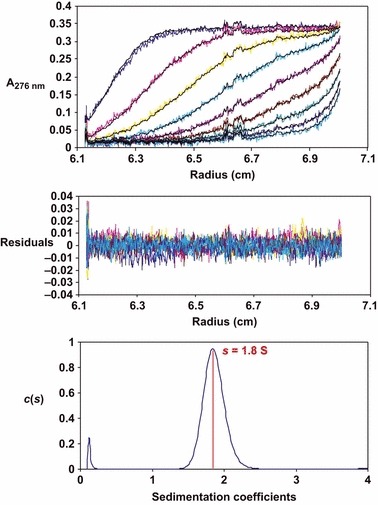
Analysis of the dispersion state of Dre2 (1–133) in solution by sedimentation velocity. Sedimentation velocity of Dre2 (1–133) was analyzed at 132 μm. Absorbance record at 276 nm versus the position within the cell (radius in cm) (upper diagram); overlay representations of the residuals (middle diagram) and sedimentation coefficient distribution [*c*(*s*)] (lower diagram) are shown.

We verified the protein oligomeric state by sedimentation velocity, a method of choice for determining the molecular weight and radius of a protein. Three different concentrations of Dre2 (1–133) (26, 132 and 660 μm) were analyzed and gave similar results. For each experimental condition, a single boundary was seen in all scans (Fig. 3, upper part), indicating the absence of higher aggregates or traces of a lower sedimentation fraction. The continuous *c*(*s*) distribution model was employed using sedfit software (http://www.analyticalultracentrifugation.com) to analyze the data. Residuals were small and randomly distributed around the zero value, indicating an excellent fit to numerical solutions of the Lamm equation (Fig. 3, middle part). The sedimentation coefficient (*S*_20,w_) distribution profile (Fig. 3, lower part) indicates that the protein sediments as a single homogeneous peak, and the *S*_0_ extrapolated at null concentration gave a value of 1.8 ± 0.1 *S* with a frictional ratio (*f*/*f*_0_) of 1.1, suggesting a spherical shape with a low hydration layer. The sedimentation velocity experiments indicate that Dre2 (1–133) is monomeric at least up to 660 μm. These conclusions were confirmed by fluorescence experiments (see below).

### The NMR structure of Dre2 (1–133) reveals a SAM methyltransferase-like fold

**Fig. 4:**
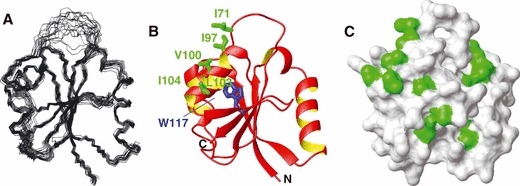
Structure of Dre2 (1–133) in solution. (A) Bundle of the final 20 best structures. (B) Ribbon representation of Dre2 (1–133). The sidechains of the residues forming an exposed semi-conserved hydrophobic patch and of the only tryptophan (W117) are indicated explicitly. The N- and C-termini are also marked. (C) CPK representation where hydrophobic residues are marked in green. The patch on helix E is particularly prominent.

We solved the structure of the isolated Dre2 (1–133) by standard NMR methods. The quality of the resulting bundle of structures is excellent, with an overall rmsd on all atoms of 1.6 Å and on the backbone atoms of 0.8 Å (Fig. 4A). The maximal dispersion of the bundle is in the loop 65–71, which mostly contains small hydrophilic residues. Residues 121–133 could not be identified in the spectrum and are therefore missing in the structure, suggesting that they are unfolded. The hydrodynamic parameters were calculated from the structure using different hydrodynamic software, giving a sedimentation coefficient of 1.6 *S* in good agreement with the analytical ultracentrifugation experiments value (1.8 ± 0.1 *S*). The domain has a very compact globular fold, which comprises a six strand β-sheet (four parallel, the other two anti-parallel) sandwiched between three helices (Fig. 4B). Two additional short helices are also present. The N- and C-termini are on the same side, separated by a β-hairpin. The presence of a histidine, an aspartate and a cysteine (residues 61, 114 and 116, respectively) relatively close in space could suggest the possibility of a heavy metal binding site, although the presence of Ile92 that intercalates between His61 and Cys116 and a lack of conservation in the family weakens the hypothesis. Several potential salt bridges stabilize the fold. Interestingly, there is a patch of exposed hydrophobic residues constituted by Ile97, Val100, Leu103 and Ile104, all on a helix, and the spatially nearby Ile71 (Fig. 4B). These residues could have functional importance and be involved in protein–protein interactions with yet unidentified partners. The only Trp117 is sandwiched between three aromatic residues (Phe73, Phe107 and Tyr115), thus accounting for the unusual appearance of the far-UV CD spectrum.

**Fig. 5:**
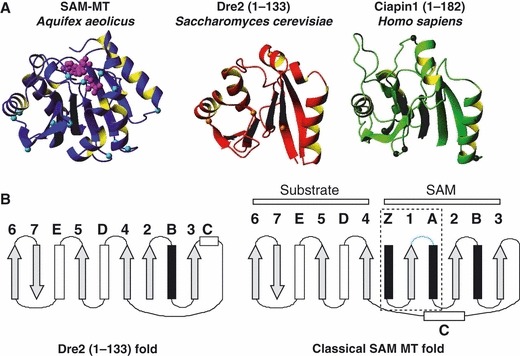
Dre2 contains a SAM-MT fold. (A) Structural comparison between Dre2 (1–133) (PDB code: 2KM1) and the two highest hits based on structural similarities in the PDB database using dali software (http://ekhidna.biocenter.helsinki.fi/dali_server/start), a SAM methyl-transferase from *Aquifex aeolicus* (PDB code: 3DH0, chain A) and the human orthologue Ciapin1/anamorsin N-terminus (1–175) (PDB code: 2YUI). SAM is indicated in pink on 3DH0. Small spheres indicate the positions of glycine residues to identify the gly-rich loop. (B) Comparison of the fold observed in Dre2 (1–133) (left) and the classical SAM methyl-transferase fold (right). The gly-rich loop is indicated in cyan. Adapted from Martin *et al.* [11].

Structure comparison against the whole Protein Data Bank (PDB) database reveals that the structure closest to the Dre2 (1–133) domain is the equivalent region of the human orthologue Ciapin1 (PDB code: 2YUI), which superposes with an rmsd of 2.5 Å and a *Z*-score of 8.2 on 106 atoms despite the very low sequence identity (12%) (Fig. 5A). The second closest hit in PDB is a SAM methyltransferase (PDB code: 3DH0, chain A) from the thermophilic bacteria *Aquifex* *aeolicus*, which superposes with an rmsd of 2.8 Å and a *Z*-score of 7.7 on 102 residues. Notably, several other methyltransferases stand also among high hits on structural similarities.

These observations suggest that the Dre2 (1–133) fold belongs to the family of SAM methyl-transferase enzymes. These enzymes usually contain alternated β-strands (β1–β7) and α-helices (αZ and αA–αE), which form a seven-stranded β-sheet forming a barrel with three helices on each side (Fig. 5B) [11]. The order of the strands is generally 3214576, with strand 7 antiparallel to the other strands. Helices Z, A and B are located on one side of the β-sheet, whereas helices C, D and E are on the other. The SAM-binding site involves poorly conserved residues in the loops following strands 1, 2 and 3, even though SAM always adopts a similar conformation. The only residues highly conserved in the SAM-binding N-terminal region of the fold are the glycine-rich sequence E/DXGXGXG between strand 1 and helix A, which interacts with the amino acid portion of SAM, and the acidic loop between strand 2 and helix B, which interacts with the ribose hydroxyls [11]. Only the loop between strand 2 and helix B is present in Dre2 (1–133). A third region is involved in the interaction with SAM: the linker between strand 4 and helix D with a hydrophobic residue forming a favourable interaction with the adenine ring of SAM [11]. The substrate-binding region, which is responsible for selectively binding small molecules, proteins, lipids or nucleic acids, is located in the highly variable C-terminal part of the β-sheet (Fig. 5B) [11]. In Dre2 (1–133), the strand order is maintained but helices Z and A and strand 1 are absent, thus forming only an open barrel. As a consequence, the glycine-rich loop is missing, suggesting that Dre2 (1–133) has retained the SAM methyl-transferase fold but might be unable to bind SAM itself. This hypothesis was supported by the fact that we were unable to detect binding of either Dre2 (1–133) or full-length Dre2 to SAM using isothermal titration calorimetry studies (data not shown).

### Characterization of the Fe-S state of Dre2

**Fig. 6:**
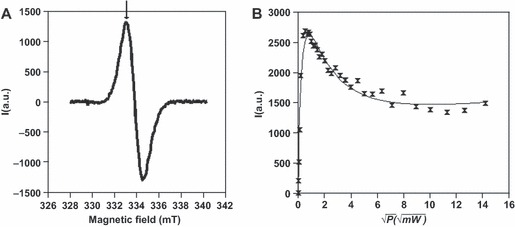
EPR measurements on Dre2. (A) Difference EPR spectrum at 20 K of a Dre2 sample (10 μm) purified anaerobically, reduced by 10 mm sodium dithionite minus the nonreduced Dre2 sample. (B) Recording of the signal versus the microwave power at the magnetic field indicated by the arrow in (A), with fitting of the intensity behaviour with the equation: 
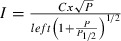
, where *I* = intensity, *P* = microwave power, *P*_½_ = microwave power at half saturation and *C* = scaling factor.

Dre2 has been described as a Fe-S cluster-containing protein, as suggested by the red–brown colour of the purified protein and further demonstrated by EPR [3]. The protein C-terminus (residues 177–348) includes eight conserved cysteines that are likely to coordinate two Fe-S clusters [3,5]. During Dre2 purification under aerobic conditions, we observed a red–brown colour by the end of the first nickel column, and a progressive loss of the colour throughout purification steps, suggesting the loss of the Fe-S clusters from the protein. Indeed, no Fe-S cluster was detectable using EPR in the final purified protein [8]. Because the absence of the Fe-S clusters could influence the protein fold, we next purified Dre2 under anaerobic conditions using an anaerobic chamber, attempting to prevent Fe-S clusters loss (see Materials and methods). By the end of the purification, the red–brown colour of the purified Dre2 protein sample was maintained stably in a sealed tube devoided of oxygen for at least 1 week. The presence of Fe-S clusters in this anaerobically purified sample was confirmed by EPR spectroscopy. Dre2 was found to be EPR-silent in accordance with the presence of [4Fe-4S]^2+^ or [2Fe-2S]^2+^ centres. Reduction of the reconstituted enzyme with sodium dithionite in excess induced the appearance of an EPR signal characterized by a *g* value at 2.03(7), typical of an *S* = 1/2 [4Fe-4S]^1+^ or [2Fe-2S]^1+^ cluster [12–15]. Figure 6A shows the EPR difference spectrum of the anaerobically purified protein (10 μm) after reduction by 10 mm sodium dithionite minus the nonreduced protein. The microwave power half-saturation (*P*_1/2_) of the Fe-S clusters EPR signal is widely used for characterizing the Fe-S cluster nuclearity. [2Fe-2S]^+^ clusters have a very low *P*_1/2_, as found in ferredoxins from spinach (*P*_1/2_ = 3.8 mW) or from maize (*P*_1/2_ = 10.5 mW) [16]. [4Fe-4S]^+^ clusters are, in contrast, characterized by a much higher *P*_1/2_ as found, for example, in the *Chromatium* high-potential iron-sulfur protein (*P*_1/2_ = 95 mW) and for ferredoxin from *Bacillus stearothermophilus* (*P*_1/2_ = 330 mW) [16]. Power saturation experiments at 20 K on the dithionite-reduced, anaerobically purified Dre2 yielded a *P*_1/2_ = 2.5 mW, indicating the presence of a [2Fe-2S]^+^ cluster (Fig. 6). Moreover, the EPR signal intensity increased when shifting temperature from 20 to 35 K, as expected for a [2Fe-2S] cluster. Spin quantitation indicated the presence of a 4 μm Fe/S cluster corresponding to 0.4 Fe/S cluster per protein. This could be partly a result of the loss of this cluster during the anaerobic purification of the protein. It is noteworthy that previously published data on Dre2 also indicate a partial occupancy of the protein by the FeS clusters [3]. The anaerobically and aerobically purified Dre2, with or without Fe-S, was used to assess influence on the Dre2 N-terminus.

### The Dre2 C-terminus and Fe-S clusters influence the flexibility of Dre2 (1–133)

We took advantage of the presence of the single tryptophan residue Trp117 of the N-terminus to analyze the influence of the C-terminus on the fluorescence properties. Monoexponentional decays, rarely observed, reflect a rigid Trp environment, whereas multi-exponentional decay signifies local protein flexibility. The fluorescence intensity decay of Trp117 of Dre2 (1–133) displays three lifetime populations, with a dominant excited state population that denotes a rigid environment, and is in agreement with the presence of this residue in the hydrophobic core of the domain. Estimating the Brownian rotational correlation time from fluorescence intensity decay yielded a value of ∼ 9 ns, compatible with that of a spherical monomeric protein of ∼ 15 kDa with an hydration radius of 40% (Table 1), which confirms the area under the curve data presented above. The same measurements performed on the full-length protein gave a Brownian rotational correlation time of ∼ 17 ns, in agreement with a monomeric state of a protein of this size (molecular weight of 38 kDa) (Table 1). Fast motions (∼ 0.2 ns) (Table 2) likely corresponding to rotations of the indole ring around the Cα-Cβ-Cγ bonds and an intermediate scale motion (nanosecond scale) reflecting some local flexibility [17–19] were observed. The wobbling-in-cone angle ω_max_ of the sub-nanosecond motion is small (19°; Table 1), indicating that the local environment around the Trp117 residue is strongly constrained.

**Table 1:** Rotational correlation time distribution of Trp117 in Dre2 (1–133) and in Fe-S cluster-bound and Fe-S cluster-free full-length (FL) Dre2

Sample	β_1_	β_2_	β_3_	θ_1_ (ns)	θ_2_ (ns)	θ_3_ (ns)	*r*_*t* = 0_	ω_max_ (°)^a^
Dre2 (1–133)	0.041 ± 0.020	0.010 ± 0.007	0.156 ± 0.002	0.17 ± 0.03	0.9 ± 0.3	8.6 ± 0.2	0.207 ± 0.020	19
Cluster-free FL Dre2	–	0.049 ± 0.007	0.132 ± 0.011	–	0.7 ± 0.2	16 ± 2	0.181 ± 0.002	27
Cluster-bound FL Dre2	–	0.034 ± 0.006	0.091 ± 0.002	–	1.1 ± 0.3	17 ± 2	0.125 ± 0.008	38

a Computed from β_3_*/r*_0_ = [1/2cos ω_max_(1 + cos ω_max_)]^2^ [36] with *r*_0_ = 0.185.

**Table 2:** Excited state lifetime distribution of Trp117 in Dre2 (1–133) and in Fe-S cluster-bound and Fe-S cluster-free full-length (FL) Dre2

Sample	α_1_	α_2_	α_3_	τ_1_ (ns)	τ_2_ (ns)	τ_3_ (ns)	<τ> (ns)^a^
Dre2 (1–133)	0.07 ± 0.05	0.13 ± 0.02	0.80 ± 0.04	0.16 ± 0.02	0.98 ± 0.5	3.18 ± 0.02	2.67 ± 0.13
Cluster-free FL Dre2	–	0.22 ± 0.02	0.78 ± 0.02	–	1.07 ± 0.14	3.30 ± 0.06	2.81 ± 0.06
Cluster-bound FL Dre2	0.18 ± 0.05	0.28 ± 0.02	0.54 ± 0.07	0.57 ± 0.22	1.59 ± 0.45	3.33 ± 0.15	2.35 ± 0.08

a Amplitude average excited state lifetime: 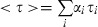.

Similar to Dre2 (1–133), the aerobically purified Fe-S cluster-free full length Dre2 exhibits a homogenous fluorescence intensity decay. By contrast, the latter is more heterogenous in the anaerobically purified Fe-S cluster-bound full-length Dre2, with a strong decrease of the longest life time population contribution to the benefit of the two shorter lifetimes (Table 2). This implies that the local flexibility of Trp117 is larger in the full-length protein containing Fe-S compared to the full-length protein devoid of Fe-S or to the isolated N-terminal domain. Similarly, the ω_max_ value also significantly increases (38°; Table 1) in the full-length Fe-S-containing Dre2, showing that the amplitude of the indole ring rotation is larger in the presence of Fe-S than that in the absence of Fe-S, or that in the absence of the C-terminus itself [Dre2 (1–133)]. Taken together, our data suggest that the presence of the Fe-S cluster in the C-terminus part of Dre2 could play a role in modulating the local flexibility of this protein.

## Discussion

*In silico* analysis of the protein sequence provides compelling evidence that the protein is formed by two regions: a globular N-terminal domain and an intrinsically unstructured C-terminus. Similar findings in the human homologue of Dre2, Ciapin1/anamorsin have been reported [20]. CD, NMR and fluorescence data confirm the *in silico* predictions and show that the N-terminus of Dre2 is a stably-folded domain, whereas the C-terminus is intrinsically unstructured, and contains eight cysteines that coordinate the Fe-S clusters. A tight interaction between the Fe-S-containing Dre2-C-terminus and the two FMN-binding and FAD-binding domains from the reductase Tah18 is essential for yeast viability [8]. This tight interaction conditions electron transfer from Tah18 to the Fe-S clusters in Dre2 and suggests that a reduced Dre2 is thus essential for yeast viability [5,8]. It is noteworthy that the Fe-S-containing Dre2 C-terminus is highly conserved through species from yeasts to humans (except in Bacteria and Archaea) but not the Dre2 N-terminus (Fig. 1B). Moreover, it is possible to remove the N-terminus domain from Dre2 without affecting yeast viability, although still severely impairing cytosolic Fe-S protein biosynthesis [8].

In an effort to characterize the Dre2 structure, we performed biochemical and biophysical analysis and finally solved the structure of Dre2 (1–133) domain by NMR. We show that this region adopts an incomplete TIM barrel structure, with six β-strands and five α-helices, and exhibits a high 3D similarity with the N-terminus from human Ciapin1. This fold belongs to the SAM methyltransferase domain superfamily, as revealed by several hits when searching for structural homologues in the PDB database [e.g. SAM-dependent methyltransferase (SAM-MT) from *Aquifex aeolicus*]. SAM-MTs share little sequence identity, despite a highly conserved structural fold [11]. Dre2 and Ciapin1 N-terminal sequences are poorly conserved (Fig. 1A), although their NMR structures are strikingly close (Fig. 5A). The SAM-MT class of enzymes includes more than 120 members, and most of them share the capacity of adding a methyl group to a substrate, using SAM as a donor. They exhibit broad substrate specificity (small molecule, lipid, protein, nucleic acid, etc.) and are involved in various biological processes. Folding prediction software suggested that the sequence of the human homologue Ciapin1 exhibits a methyltransferase domain at its N-terminus, although purified Ciapin1 did not prove any measurable DNA or RNA methyltransferase activity [21]. Interestingly, a subclass of SAM-MT has been recently characterized in which SAM is used to generate an oxidizing agent, the 5′-deoxyadenosyl radical, from a [4Fe-4S]^+^ cluster of the protein, serving as reducing agent. This class of enzyme, known as the radical SAM superfamily [22], is involved in diverse biological pathways as a result of being capable of complex chemistry, leading to the generation of a highly reactive intermediate 5′-deoxyadenosyl radical SAM. All known examples of SAM radical enzymes exhibit complete (βα)_8_ or partial (βα)_6_ TIM barrels. Dre2 (1–133) shares the latter feature with a partial barrel (β_6_α_5_). However, because it lacks the first three elements of secondary structure that comprise a glycine-rich loop involved in SAM binding, it is possible that Dre2 is a divergent member of the family. Whether Dre2 retains the function of a radical SAM-MT remains to be determined. SAM-binding N-terminal region, which includes the highly conserved residues E/DXGXGXG, is also lacking in a Ciapin1 N-terminal region. Accordingly, SAM was shown not to bind this region *in vitro* [20]. The binding of SAM appears to be controlled in radical SAM reactions as a means of preventing uncoupled cleavage of SAM in the absence of substrate [23]. For example, SAM binding has been shown to be increased by more than 20-fold compared to biotin synthase in the presence of its substrate dethiobiotin [24]. The presence of a yet unidentified partner might be necessary to confirm SAM binding to Dre2 or Ciapin1 *in vitro*.

Our data provide an insight into the structure–function relationship of Dre2. The presence of the C-terminus of the protein Dre2 influences local motions so that the local flexibility is increased in the full-length protein. By extension, a modulation of the N-terminus activity by the C-terminus is thus possible. It has been suggested also that Dre2 might be trapped in the inter-membrane space of mitochondria as a result of disulfide bond formation between cysteine residues via the Mia40/Erv1 system [3]. This phenomenon might also influence the N-terminus. Structural knowledge suggests that functional regions could include a semi-conserved and exposed hydrophobic patch on helix E (96–105) and might be involved in the recognition of a yet unidentified partner. It is noteworthy that the N-terminal 1–152 residues of Dre2 are necessary for normal cytosolic Fe-S cluster biosynthesis [8]. In accordance with previously reported data involving Dre2 in cytosolic Fe-S protein biogenesis [3], it is possible that those exposed residues are involved in the recognition of a yet unknown substrate of Dre2. Although much remains to be investigated with respect to elucidating the role of Dre2, our data provide an important step forwards in understanding the molecular function of Dre2.

## Materials and methods

### *In silico* analysis

Sequences for multiple alignments were recovered by a blast search. The alignment was performed using muscle software [25]. Dre2 sequences were analyzed using pondr software [26] and foldindex [27] for folding prediction. A search for sequence motifs or sites was performed using interproscan (http://www.ebi.ac.uk/Tools/pfa/iprscan/). For phylogenetic analysis, homologous sequences were recovered by blast searches in a public nonredundant database (http://www.ncbi.nlm.nih.gov/), and multiple alignment was performed with the selected sequences using muscle software [25]. Only homologous positions were used to build a maximum likelihood tree using phyml [28]. The JTT model of amino acid substitution was chosen, and a gamma correction with four discrete classes of sites was used. The α parameter and the proportion of invariable sites were estimated. The robustness of the tree was tested by nonparametric bootstrap analysis using phyml.

### *E. coli* over-expression and purification of Dre2 (1–133)

Over-expression and purification of Dre (1–133) was performed as described previously [8]. At the end of the procedure, Dre2 (133) was recovered in the flowthrough and protein purity was estimated by SDS/PAGE.

### Aerobic and anaerobic purification of full-length Dre2

Overexpression of full-length Dre2 was performed in the presence of 125 μm ferric-nitrilotriacetate for 5 h at 18 °C. Aerobic purification of full-length Dre2 was performed at 4 °C, as described previously [8]. Purification under anaerobic conditions was performed in a COY anaerobic chamber. All fractions were recovered and stored on ice. All buffers were degassed and 2 mm dithionite was added at each step to prevent protein oxidation. Lysis and TEV digestion were performed in the anaerobic chamber, although sonication and ultracentrifugation were performed aerobically. After elution from a DEAE-sephacel column, fractions containing Dre2 were recovered out of the anaerobic chamber and quickly frozen in liquid nitrogen and stored at −80 °C.

### EPR

EPR experiments were performed at 20 K on a Bruker Elexsys 500 spectrometer (Bruker, Ettlingen, Germany) with an SHQ001 cavity fit and an ESR900 cooling system. For power saturation study at 20 K, a 2D experiment was performed by measuring intensity against the magnetic field at different microwave powers from 200 to 0.0002 mW using modulation amplitude 1 mT, field modulation frequency 100 kHz, time constant 0.01024 s and microwave frequency 9.44342 GHz. Because of the low signal/noise ratio of the EPR spectra of the anaerobically purified protein (10 μm) in the presence of sodium dithionite (10 mm), the difference spectra of the dithionite-reduced anaerobically purified protein minus the nonreduced protein were considered. Spin quantitation was carried out under nonsaturating conditions using 15 μm Cu(II)-ethylene diamine tetraacetate as a standard.

### CD

CD spectra were performed with a Jasco 715 spectropolarimeter (Jasco Inc., Easton, MD, USA) equipped with a Peltier temperature control unit. Far-UV spectra were recorded in the range 195–260 nm with 10 μm Dre2 (1–133) in 20 mm sodium phosphate, 100 mm NaCl, 1 mm Tris(2-carboxyethyl)phosphine (pH 7.5) at 10 °C using a 0.1-cm quartz cell. An average of 50 scans at 100 nm·min^−1^ (response time of 2 s) was recorded. Near-UV spectra (four scans) were monitored in the range 250–320 nm with 45 μm Dre2 (1–133) in 25 mm sodium phosphate (pH 7.5), 20 mm NaCl and 2 mmβ-mercaptoethanol at 10 °C. Thermal denaturation curves were performed at 222 nm and at 291.8 nm from 10 to 80 °C (1 °C·min^−1^). For all experiments, the buffer spectrum was subtracted from the sample spectra.

### Analysis for assessing monodispersion of Dre2 (1–133)

For sedimentation velocity, Dre2 (1–133) samples (660, 132 and 26 μm, respectively) were centrifuged in a Proteomelab XL-I analytical ultracentrifuge (Beckman Coulter, Fullerton, CA, USA) at 25 °C in a four hole rotor AN60-Ti equipped with 3-mm (660 μm) or 12-mm double-sector cells with epon centrepieces. Detection of the protein concentration as a function of radial position and time was performed by measuring *A*_247_ (660 μm) or A_276_. Dre2 (1–133) samples were spun in 50 mm sodium phosphate at pH 6.8, 100 mm NaCl, 1 mm dithiothreitol for 16 h at 42 000 r.p.m. Parameters were calculated using sednterp, version 1.09 (http://www.jphilo.mailway.com/download.htm) and used for the analysis of the experiment: partial specific volume 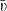 = 0.753 mL·g^−1^, viscosity η = 0.009162 P and density ρ = 1.00568 g·mL^−1^. Sedimentation velocity data analysis was performed by continuous size distribution analysis *c*(*s*) using sedfit, version 11.8 [29]. All the *c*(*s*) distributions were calculated with a fitted frictional ratio *f/f*_*0*_ and a maximum entropy regularization procedure with a confidence level of 0.95. Theoretical sedimentation values of Dre2 (1–133) were generated using hydropro 7c [30] with a 2.2 Å for the radius of the atomic elements and us-somo [31].

### NMR spectroscopy

NMR spectroscopy was carried out at 25 °C and either 500 or 600 MHz on 0.5 mm samples of Dre2 (1–133) in 50 mm sodium phosphate at pH 6.8, 1 mm dithiothreitol and 100 mm NaCl. Virtually complete spectral assignment was achieved using 2D DQF-COSY, ^1^H-^1^H NOESY, ^1^H-^1^H TOCSY and ^1^H-^15^N HSQC and 3D ^1^H-^15^N NOESY-HSQC, ^1^H-^15^N TOCSY-HSQC, HNCO; HN(CA)CO, HNCA, HN(CO)CA, HN(CA)CB, ^1^H-^13^C NOESY and ^1^H-^13^ HCCH-TOCSY.

The only residues that could not be identified in the spectra correspond to residues G-2, H-1 and M0, which are a tag introduced for cloning purposes, and residues 126–133, likely because of exchange with the solvent. Residues K124 and L125 could be only partially assigned. The assignment has been deposited in the BMRB database (entry number 18235).

Structure calculations were carried out by simulated annealing using standard protocols for discover i2005 and the consistent valence force field (Accelrys, San Diego, CA, USA). Starting from an initial extended conformation, 200 structures were generated using simulated annealing, including a 30-ps high-temperature phase (at 1000 K), followed by a cooling phase (down to 300 K) of 8 ps and final energy minimization. A force constant of 20 kcal·mol^−1^·Å^−2^ was used for NOE distance restraints. Restraints of dihedral Φ and Ψ angles used a force constant of 30 kcal·mol^−1^·rad^−2^. After initial testing to clean the data from inconsistencies, 200 final structures were calculated. The quality of the final structures, selected according to the potential energy and the compatibility with experimental restraints, was analyzed using the whatif (http://swift.cmbi.kun.nl/whatif/) and procheck-nmr (http://www.biochem.ucl.ac.uk/~roman/procheck_nmr/manual/manprochint.html). The 20 best structures in terms of energy were deposited in the PDB under accession number 2KM1.

### Fluorescence spectroscopy

Intrinsic tryptophan fluorescence was measured at room temperature using a Jasco FP777 spectrofluorimeter, with a 295 nm excitation and a band-pass of 3 nm, low PMT gain and scan speed of 100 nm·min^−1^. The emission spectrum was obtained with a band-pass of 10 nm, and was recorded between 300 and 400 nm. Dre2 (1–133) (1 μm) was in 25 mm sodium phosphate (pH 7.5), 20 mm NaCl and 2 mmβ-mercaptoethanol.

### Time-resolved fluorescence measurements

Fluorescence intensity and anisotropy decays were measured by the time-correlated single-photon counting technique from the polarized *I*_vv_(*t*) and *I*_vh_(*t*) components. A light-emitting diode (PLS 295, serial number PLS-8-2-237; Picoquant, Berlin-Adlershof, Germany) (maximal emission at 298 nm) working at 10 MHz was used as an excitation source and a Hamamatsu photomultiplier (model R3235-01; Hamamatsu Corp., Bridgewater, NJ, USA) was used for detection. The LED emission, focused with a UV lens, was filtered through a short-pass Asahi Spectra UV filter ZUS300 (Asahi Spectra USA Inc., Torrance, CA, USA). Fluorescence emission was collected through a 306AELP Omega long-pass filter (Omega Optical, Brattleboro, VT, USA) and a UG11 Schott broad-band glass filter (UQG Optics Ltd, Cambridge, UK). The instrument response function was obtained at the excitation wavelength, with a glycogen scattering solution. As previously described, fluorescence intensity *I*(t) and anisotropy decays *r*(*t*) were analyzed as sums of 150 or 100 exponential terms, respectively, by the maximum entropy method [32,33] according to the equations: *I*(*t*) = ∑α_i_exp(−*t*/τ_i_) where α_i_ is the normalized amplitude and τ_i_ is the excited state lifetime, and *r*(*t*) = ∑β_i_exp(−*t*/θ_i_) where β_i_ is the anisotropy and θ_i_ is the rotational correlation time. In this analysis, we assume that each lifetime τ_i_ is associated with all rotational correlation times θ_I_ [34]. The Skilling–Jaynes entropy *S* was subjected to a χ^2^ constraint [35] to ensure that the recovered distribution would be consistent with the data.

## Abbreviations

Ciapin1: cytokine-induced apoptosis inhibitor 1
PDB: Protein Data Bank
SAM: *S*-adenosylmethionine
SAM-MT: SAM-dependent methyltransferase

## Supporting information

The following supplementary material is available:

**Fig. S1.** Purification of the Dre2 N-terminus.

This supplementary material can be found in the online version of this article.

Please note: As a service to our authors and readers, this journal provides supporting information supplied by the authors. Such materials are peer-reviewed and may be re-organized for online delivery, but are not copy-edited or typeset. Technical support issues arising from supporting information (other than missing files) should be addressed to the authors.
