# Supplementary material for: A S-adenosylmethionine methyltransferase-like domain within the essential, Fe-S-containing yeast protein Dre2
Source: FEBS J. 2012 Jun;279(12):2108–19. doi: 10.1111/j.1742-4658.2012.08597.x (PMC3440578; doi:10.1111/j.1742-4658.2012.08597.x)
Supplement: Supplementary file 1 [file febs0279-2108-SD1.pdf]

the FEBS Journal

www.febsjournal.org

A *S*-adenosylmethionine methyltransferase-like domain within the essential, Fe-S-containing yeast protein Dre2

Nicolas Soler, Constantin T. Craescu, Jacques Gallay, Yves-Michel Frapart, Daniel Mansuy, Bertrand Raynal, Giuseppe Baldacci, Annalisa Pastore, Meng-Er Huang, and Laurence Vernis

DOI: 10.1111/j.1742-4658.2012.08597.x

Figure S1 (related to Figure 1)

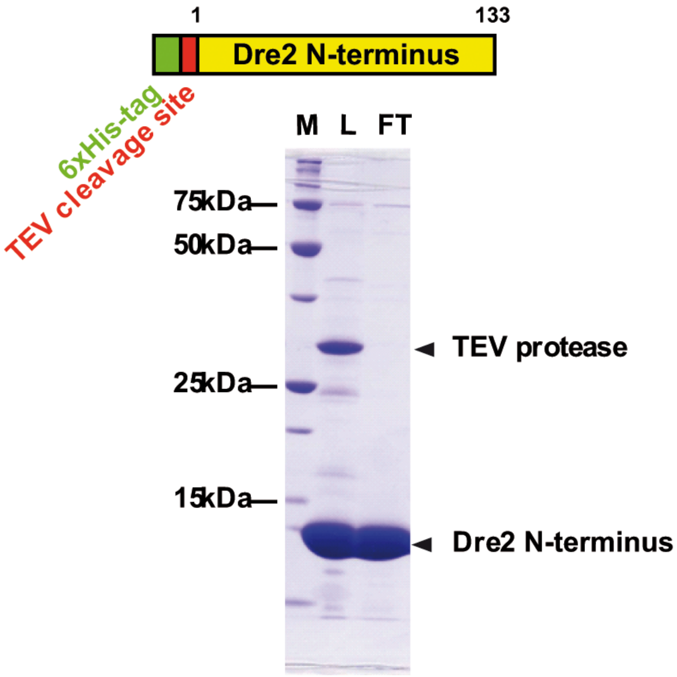

<u>Figure S1: Purification of Dre2 N-terminus</u>

<u>Legend of figure S1 - Schematic drawing of Dre2 1-133 construct fused to a 6xHis-tag (green box) and a TEV cleavage site (red box) and SDS-PAGE analysis of Dre2 (1-133) after the final elution step of purification.</u>

M = molecular weight size Marker, L= Load on the second NiNTA column after TEV digestion, FT= Flowthrough, after the second NiNTA column (estimated purity >95%).
